# Completeness and usability of ethnicity data in UK-based primary care and hospital databases

**DOI:** 10.1093/pubmed/fdt116

**Published:** 2013-12-08

**Authors:** Rohini Mathur, Krishnan Bhaskaran, Nish Chaturvedi, David A. Leon, Tjeerd vanStaa, Emily Grundy, Liam Smeeth

**Affiliations:** 1 Department of Non-Communicable Disease Epidemiology, London School of Hygiene and Tropical Medicine, London WC1E 7HT, UK; 2 NHLI Division, Faculty of Medicine, International Centre for Circulatory Health, London W2 1LA, UK; 3 Utrecht Institute for Pharmaceutical Sciences, Utrecht University, Utrecht TB 3508, The Netherlands; 4 Medicines and Healthcare Products Regulatory Agency, LondonSW1W 9SZ, UK; 5 Department of Social Policy, London School of Economics and Political Science, London WC2A 2AE, UK

**Keywords:** epidemiology, ethnicity, methods

## Abstract

**Background:**

Ethnicity recording across the National Health Service (NHS) has improved dramatically over the past decade. This study profiles the completeness, consistency and representativeness of routinely collected ethnicity data in both primary care and hospital settings.

**Methods:**

Completeness and consistency of ethnicity recording was examined in the Clinical Practice Research Datalink (CPRD) and Hospital Episode Statistics (HES), and the ethnic breakdown of the CPRD was compared with that of the 2011 UK censuses.

**Results:**

27.1% of all patients in the CPRD (1990–2012) have ethnicity recorded. This proportion rises to 78.3% for patients registered since April 2006. The ethnic breakdown of the CPRD is comparable to the UK censuses. 79.4% of HES inpatients, 46.8% of outpatients and 26.8% of A&E patients had their ethnicity recorded. Amongst those with ethnicity recorded on >1 occasion, consistency was over 90% in all data sets except for HES inpatients. Combining CPRD and HES increased completeness to 97%, with 85% of patients having the same ethnicity recorded in both databases.

**Conclusions:**

Using CPRD ethnicity from 2006 onwards maximizes completeness and comparability with the UK population. High concordance within and across NHS sources suggests these data are of high value when examining the continuum of care. Poor completeness and consistency of A&E and outpatient data render these sources unreliable.

## Introduction

The capture of ethnic group information in routine health records is recognized in the UK as a necessary pre-requisite to addressing inequalities in health service usage and outcomes.^1–4^ Although the facility to record ethnicity was introduced into primary care in 1991 and into the Hospital Episode Statistics (HES) for England in 1995, unsystematic implementation resulted in poor completeness and quality of the initial data, limiting their usefulness for clinical care, commissioning and research.^5–10^

Within primary care, the incentivization of ethnicity recording under the Quality and Outcomes Framework (QOF)^11–13^ between 2006/07 and 2011/12 dramatically improved the completeness of ethnicity data for newly registered patients. QOF results data show that over 90% of UK general practices are now recording ethnicity for all of their newly registered patients.^14^ Similarly, high levels of recording have recently been reported for hospital inpatients in England.^9^

The Clinical Practice Research Datalink (CPRD, formerly the General Practice Research Database) is a primary care research database which collates anonymized patient data from 624 general practices across the UK, covering 6.3% of all 9949 practices in 2012. Similarly, the HES for England comprised data on all inpatient, outpatient and accident and emergency admissions to National Health Service (NHS) hospitals in England.^15–22^

Although the validity of morbidity indicators in both sources has been explored in depth, no comprehensive audit of ethnicity data has been conducted.^15,23,24^ Furthermore, even though improvements in ethnicity recording have been demonstrated nationally, very few epidemiological studies have utilized the patient level ethnicity data available.^25^ Confirming that the ethnicity data recorded in routine health records are of sufficient quality to explore ethnic differentials in health is an essential first step towards maximizing their use for research and clinical purposes.^26^ As such, the aims of this study are threefold: to examine the completeness of ethnicity recording over time and assess consistency for individuals whose ethnicity is recorded multiple times in both the CPRD and HES; to compare the ethnic breakdown of the CPRD population with that of the 2011 UK Census and finally, to examine the extent to which ethnicity is matched or discrepant for patients contributing to both CPRD and HES and to generate recommendations for future use of these data.

## Methods

### Data extraction

#### CPRD (1987–2012)

Ethnicity in the CPRD is coded using the Read system of alphanumeric codes.^27^ Read codes for ‘Ethnic Group’ falling under the 9i (2001 Census) and 9S (1991 Census) hierarchies were extracted for all current and past patients contributing to the July 2012 build of the database. All ethnicity codes were collapsed into the 16 categories of the 2001 Census (see Supplementary data). Usable ethnicity was considered to be any 9i or 9S Read code, which is not unknown (9SD, 9SE, 9SZ, 9iG), at too high a level to be interpreted (9i, 9S), or missing.

#### HES (1997–2012)

In August 2012, all ethnicity and demographic data for patients contributing to the HES for England were extracted. All codes were collapsed into the 16 categories of the 2001 census. Usable ethnicity was considered to be any ethnic code which is not unknown (ethnos codes 9, X, Z), or missing. Since the unique patient identifier ‘hesid’ has only been used since 1997 onwards, inpatient data for the financial years 1997/08–2011/12 were included in the analysis. Outpatient data were available from 2003 to 04 onwards and accident and emergency (A&E) data from 2007 to 08 onwards.

### Statistical analysis

#### Overall completeness of ethnicity recording

In both databases, the proportion of patients with ethnicity ever recorded was calculated. For CPRD completeness was compared between (i) all patients including those who have left or died, (ii) currently registered patients (that is all patients who have not died or transferred out of their general practice) and (iii) patients registered after 1 April 2006 when incentivization of ethnicity recording was introduced to primary care. For HES, completeness was assessed for all inpatients, outpatients and A&E attendees.

In CPRD, ethnicity recording was further broken down by year of first ever registration. For HES, ethnicity recording was further broken down by year of first ever admission (inpatients and A&E) or appointment (outpatients).

#### Multiple ethnicity recording within sources

In both general practice and in hospital, patients can have their ethnicity recorded repeatedly over multiple consultations or visits. Discrepancies may arise if there are mistakes while entering the data or if the patient chooses a different ethnic group when asked by the service provider.

In order to examine consistency of ethnicity coding the proportion of patients with only one ethnicity code on their record was compared with the proportion of patients with multiple codes which could be:

- truly matched (multiple ethnic codes which are identical);
- categorically matched (multiple ethnic codes which are different but fall into the same five higher-level groups of ethnicity, namely White, Mixed, Asian/Asian British, Black/Black British, Chinese/Other);
- truly mismatched (multiple ethnicities which span different higher level groups).

#### Discrepant ethnicity recording between linked sources

Of the 624 general practices contributing to the CPRD, 357 were linked with HES. Linkage using deterministic matching on NHS number, date of birth and gender was undertaken by a Trusted Third Party. For the 561 602 patients registered from 1 April 2006 onwards with a valid ethnicity recorded in *both* CPRD and HES, we compared the most commonly recorded ethnicity code in each database to determine the proportion of patients with matched or mismatched ethnicity across databases. The degree of mismatch was further examined for each ethnic group in turn.

#### Comparison of the CPRD population with the 2011 UK census population

The most recent census across the UK was undertaken on 27 March 2011. Ethnic breakdowns for the populations of England, Wales, Scotland and Northern Ireland were obtained from the relevant census websites. Since the available categories for the ethnicity question vary slightly between the censuses for the constituent countries, categories were collapsed for comparison with the CPRD data (see Supplementary data).

The ethnic breakdown of the census population was compared with that of all CPRD patients who were actively registered on 27 March 2011. The most recent ethnicity code prior to the census date was collapsed into the five higher level categories of the 2011 Census for analysis. The proportion of patients belonging to each ethnic group in the CPRD was then compared with the 2011 census. For comparison, the crude proportions were directly age standardized against the age distribution of the 2011 Census.

## Results

### Overall completeness of ethnicity recording

#### CPRD population

From a total of 12 099 672 patients contributing to the July 2012 build of the CPRD (including patients who have died or left the practice), 3 544 589 patients (29.3%) had at least one Read code for ethnicity, including unusable codes (not stated, not known, at too high a level to be usable). Patients registered from April 2006 onwards comprised 18.2% of the whole database. The proportion of patients with at least one usable ethnicity code recorded ranged from 27% for the whole of CPRD to 76% for patients registered from 2006 onwards (Table 1).

**Table 1.** Overall completeness of ethnicity recording in CPRD (July 2012)

	*All acceptable patients*		*Currently registered*		*Registered 1 April 2006 onwards*	
	*n*	*%*	*n*	*%*	*n*	*%*
Number of patients (%)	12 099 672	100.0	5 308 411	100.0	2 201 065	100.0
% with any ethnicity recorded (including not stated, not known)	3 544 589	29.3	2 605 232	49.1	1 874 916	85.2
% with usable ethnicity recorded (excluding not stated, not known)	3 282 739	27.1	2 423 438	45.7	1 723 195	78.3
% with only 1 ethnicity on their record	2 802 284	23.2	2 038 097	38.4	1 481 112	67.3
% with multiple ethnicities on their record	480 455	4.0	385 341	7.3	242 083	11.0
% with multiple ethnicities which are truly identical	379 591	3.1	306 771	5.8	180 455	8.2
% with multiple ethnicities which are categorically identical	62 413	0.5	49 144	0.9	35 208	1.6
% with truly discrepant ethnicity	38 523	0.3	39 426	0.7	26 420	1.2
% with no usable ethnicity on their record	8 816 933	72.9	2 854 973	53.8	486 870	22.1

**Fig. 1.**
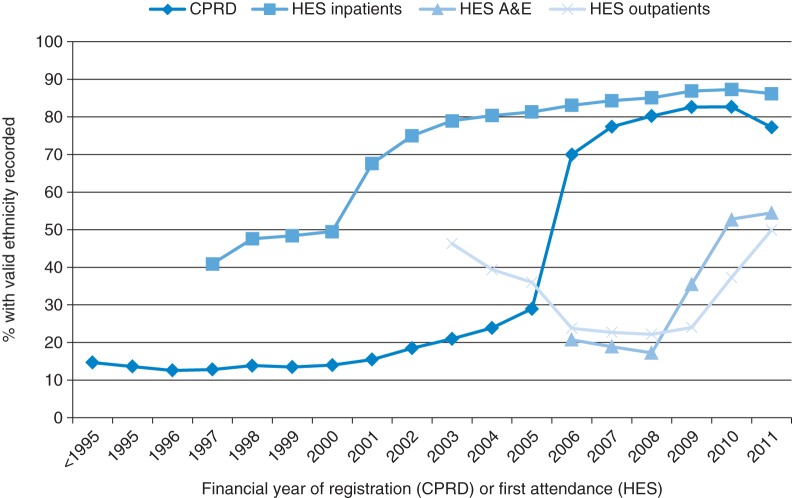
Proportion of patients with valid ethnicity recorded in CPRD and HES by financial year of registration (CPRD) or first attendance (HES).

Figure 1illustrates the stark contrast in completeness before and after April 2006, when ethnicity recording became financially incentivized under the QOF. The completeness of ethnicity recording reached 90% in 2010 across general practices in the CPRD. We found no notable differences by gender. However, ethnicity recording was consistently highest for patients aged 40–79 in all years. Recording was markedly lower for patients aged 80 and over, though this gap diminished over time (see Supplementary data).

#### HES population

All patients in HES had an ethnicity code attached to every attendance or episode. As such, the completeness of ethnicity recording, including unusable codes, was 100% for all three data sets, though the proportion of patients with usable ethnicity varied significantly between them (Table 2).

**Table 2.** Overall completeness of ethnicity recording in HES (April 2012)

	*Inpatients*		*Outpatients*		*A&E*	
	*n*	*%*	*n*	*%*	*n*	*%*
Number of patients (%)	51 965 028	100	48 549 620	100	31 860 530	100
% with any ethnicity recorded (including not stated, not known)	51 965 028	100	48 549 620	100	31 860 530	100
% with usable ethnicity recorded (excluding not stated, not known)	41 281 350	79.4	17 696 595	36.5	8 531 890	26.8
% with only 1 ethnicity on their record	16 354 201	31.5	4 608 411	9.5	5 860 016	18.4
% with multiple ethnicities on their record	24 927 146	48.0	13 088 173	27.0	2 671 874	8.4
% with multiple ethnicities which are truly identical	22 883 676	44.0	2 589 948	5.3	2 620 117	8.2
% with multiple ethnicities which are categorically identical	652 246	1.3	34 697	0.1	7 740	0.2
% with truly discrepant ethnicity	1 391 227	2.7	10 463 828	21.6	44 287	0.1
% with no usable ethnicity on their record	10 683 678	20.6	30 853 025	63.5	23 328 640	73.2

#### HES inpatient population

A total of 51 965 028 patients contributing 223 451 171 inpatient episodes across 14 years (1997/08–2011/12) were available for analysis. 66.5% of patients had at least one code which was usable. The proportion of patients with usable ethnicity improved from 41% for patients who were first admitted in 1997 to 86% for patients who were first admitted in 2011 (Fig. 1).

#### HES accident and emergency population

A total of 31 860 530 patients contributed 73 085 977 A&E visits across 5 years (2007/08–2011/12. 26.8% of these patients had a code which was usable. The proportion of patients with usable ethnicity recorded improved from 20% for patients who were first admitted in 2008 to 53% in for patients who were first admitted in 2011 (Fig. 1).

#### HES outpatient population

In total, 48 549 620 patients contributed 574 625 389 outpatient appointments over 8 years (2003/04–2011/12). 36.5% of patients had a code which was usable. The proportion of patients with usable ethnicity recorded did not improve markedly over time, rising from 46% in 2003 to 50% in 2011, with completeness falling to a low of 22% in the interim (Fig. 1).

### Multiple ethnicity recording within sources

#### CPRD population

Within the whole of CPRD, 4.3% of patients have had their ethnicity recorded multiple times. This increased to 7.3% for patients who were currently registered, and to 11.0% for patients registered from April 2006 onwards. Amongst patients with multiple ethnicity codes, the proportion with codes either truly identical (at the 16 group level) or the same in aggregate (at the 5 category level) was consistently high, ranging from 89.1% for the 2006+ population, to 92.0% for the currently registered and total populations (Table 1).

#### HES population

Multiple ethnicities were recorded for 48% of inpatients, 27% of outpatients and 8.4% of A&E patients. Amongst patients with multiple ethnicity codes, codes were either truly or categorically identical for 94.4% of inpatients and 98.4% of A&E patients and 20.1% for inpatients (Table 2).

### Missing and discrepant ethnicity in linked sources

In total, 827 753 patients in the July 2012 build of the CPRD database registered from 1 April 2006 onwards also had linked HES data available. Completeness of usable ethnicity was 78.7% for CPRD, 86.3% for HES and 97.1% for the combined database.

To examine matched and mismatched ethnicity between linked CPRD and HES, the analysis was restricted to 561 602 (67.8%) patients with a usable ethnicity code in both databases. When comparing the most commonly recorded ethnicity, 72.7% of patients had an ethnicity code which belonged to the same 16-level category in both databases. This proportion increased to 85.0% when collapsing the 16 categories into five groups.

When exploring the agreement between specific ethnic groups, we found the direction of mismatch to be similar going from both CPRD→ HES and from HES→CPRD. Over 96% of individuals coded as White in one database were White in the linked database. Almost half of all individuals coded as South Asian in CPRD were South Asian in HES. Conversely, 71% of South Asians in HES were coded the same in CPRD. Patients coded as Black in CPRD were most commonly coded as Mixed in HES. Individuals coded as Mixed in CPRD were most commonly White in HES; however, most individuals coded as Mixed in HES were coded as Black in CPRD. Individuals coded as Other in CPRD were most commonly coded as South Asian or Other in HES; most individuals coded as Other in HES were coded as White in CPRD (Table 3).

**Table 3.** Proportion of patients with matching ethnicity in linked CPRD and HES (*n* = 561 502)

*Most common CPRD ethnic group*	*Most common HES ethnic group*						*Total*
	*White*	*South Asian*	*Black*	*Other*	*Mixed*	*Equally common*	
White	453 244	1294	2082	9549	2095	2271	470 535
Row%	96.33	0.28	0.44	2.03	0.45	0.48	100
Column%	97.66	5.17	30.76	45.47	5.36	40.88	83.8
South Asian	1 545	17 636	498	3 256	11 619	1154	35 708
Row%	4.33	49.39	1.39	9.12	32.54	3.23	100
Column%	0.33	70.51	7.36	15.5	29.74	20.77	6.36
Black	1447	452	1136	1645	21 873	822	27 375
Row%	5.29	1.65	4.15	6.01	79.9	3	100
Column%	0.31	1.81	16.78	7.83	55.99	14.8	4.88
Other	2804	4192	271	3392	677	495	11 831
Row%	23.7	35.43	2.29	28.67	5.72	4.18	100
Column%	0.6	16.76	4	16.15	1.73	8.91	2.11
Mixed	3487	770	2418	2237	1754	621	11 287
Row%	30.89	6.82	21.42	19.82	15.54	5.5	100
Column%	0.75	3.08	35.73	10.65	4.49	11.18	2.01
Equally common	1572	667	363	923	1049	192	4766
Row%	32.98	13.99	7.62	19.37	22.01	4.03	100
Column%	0.34	2.67	5.36	4.39	2.69	3.46	0.85
Total	464 099	25 011	6768	21 002	39 067	5555	561 502
Row%	82.65	4.45	1.21	3.74	6.96	0.99	100
Column%	100	100	100	100	100	100	100

### Comparison of the CPRD population with the 2011 UK census population

From the 12 099 672 patients contributing to the July 2012 build of the CPRD database, 5 219 411 were active on census day, 27 March, 2011. Within this population, 1 446 254 had registered on or after 1 April 2006.

#### Region, age and gender comparison

Compared with the 2011 Census, the CPRD population has a slightly higher proportion of individuals from Scotland, Wales and Northern Ireland, and a smaller proportion of individuals residing in England. The age structure of the active CPRD population on census day is virtually identical to that of the UK census population, indicating that patients contributing to CPRD are representative of the UK population in terms of age. Registrations from 2006 onwards relate to a much younger population, as would be expected as this population excludes some older individuals, but includes all children born from April 2006 onwards (see Supplementary data).

#### Ethnicity comparison

**Fig. 2.**
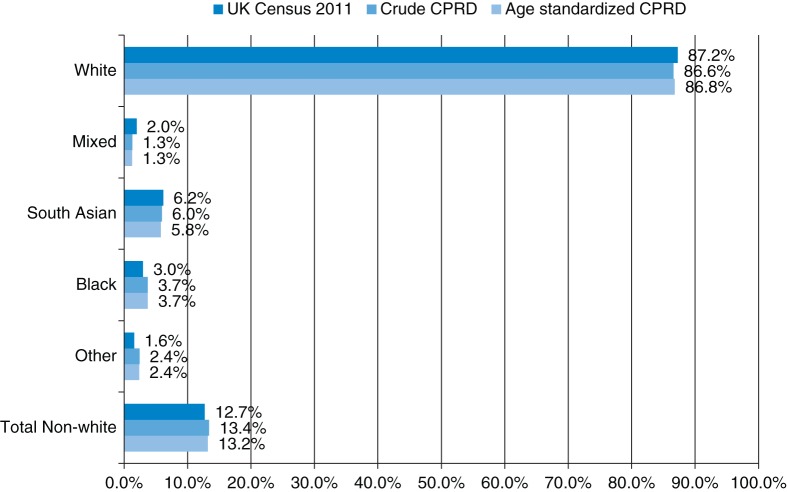
Ethnic breakdown of CPRD and UK population on 27 March 2011.

42% of the CPRD patients analysed had ever had their ethnicity recorded. The ethnic breakdown of the UK population in the 2011 census is very similar to the whole CPRD population on that date, both before and after age standardization (Fig. 2).

## Discussion

The relationship between ethnicity and health is complex, in ways we are only beginning to understand. Good quality data are critical for researchers to fully understand how ethnicity relates to a wide range of health outcomes, particularly long-term conditions with complex causal mechanisms such as diabetes and stroke. Furthermore, as the ethnic minority population of the UK is, on average, younger than the White British population, ethnicity data are of vital importance in predicting the burden of disease which is yet to peak in these population groups, and for allocation of health resources and infrastructure. Large routine healthcare data sets are uniquely valuable, in that they offer sufficient power to study individual ethnic groups, gender differences and trends across generations, which would be unfeasible with *de novo* cohorts.

### Main findings of this study

This study compared the completeness of self-reported ethnicity recording in a sample of UK-wide primary care patients and complete records from English secondary care. It further investigated the generalizability of primary care ethnicity data by comparing the ethnic breakdown of the CPRD with that of the UK census, and explored issues of multiple and discrepant recording both within each resource and across linked databases. The study showed that, as of 2010, valid ethnicity is now being recorded for 90% of newly registered patients in primary care, 77% of HES inpatients and 50% of both HES A&E patients and outpatients.

Over 80% of patients in CPRD and 90% of HES inpatients and A&E patients with multiple ethnicities had codes which are either truly identical, or fell into the same five high-level groups. However, the recording of ethnicity for HES outpatients was highly inconsistent, limiting the usability of this particular data set.

The ethnic breakdown of the CPRD, which has been shown to be representative of the UK population in terms of age and gender, was found to be comparable to that of the combined censuses for England, Wales, Scotland and Northern Ireland.

The overall completeness of ethnicity recording is comparable to that of other UK-based primary care databases such as QRESEARCH (33.5%)^28^ and The Health Improvement Network (23.1%).^29^ Furthermore, the completeness of *usable* ethnicity coding shown here for HES inpatients is comparable to that found by Jack *et al.* in 2002/3 (81.1%) and Mindell *et al.* in 2003/4 (79%).^20,30^

Linkage of the CPRD and HES inpatient data improved completeness to 88.9% overall and to 97.1% for those registered from 2006 onwards. The benefits of database linkage on reducing missing data have previously been detailed in a study linking the UK Renal Registry to HES inpatient data, and Office for National Statistics Mortality data. Similar to this study, the authors found completeness of ethnicity recording improved from 75.5% in the UKRR to 98.9% after linkage.^31^

Finally, though agreement between HES and CPRD were found to be high overall, this was driven primarily by patients coded as being of White ethnicity. For patients of South Asian ethnicity, the agreement was only 50%, and weaker still for other ethnic groups. The findings here mirror those of a recent study which compared ethnicity recorded in HES to the ‘gold standard’ of self-reported ethnicity as captured in the 2010 Cancer Patient Experience Survey in England. The study reported high concordance of HES coding for patients of White British ethnicity, but far weaker agreement for all other ethnic groups.^32^

### What is already known on this topic

We know that routinely collected ethnicity data in UK-based healthcare databases is underutilized for observational epidemiological studies.^25^ Reasons for this include perceptions of poor completeness and quality of these data. National programmes targeting the improvement of this measure have been implemented, but current completeness and usability of these data have not been previously audited.

### What this study adds

This study has demonstrated that ethnicity is being captured for the majority of the population in routine electronic healthcare records, and that these data are largely complete and comparable to the general population. Linkage of data sets yields completeness of almost 100%, with high levels of agreement for patients of White ethnicity, but poorer for ethnic minority groups.

Previous studies have ascribed patient ethnicity indirectly via name-recognition software or by estimating ethnic population size from census data. Both these methods are of questionable validity, particularly for individuals of mixed ethnicity and for descendents of migrants.^33–36^ Though these methods have been useful for certain situations in the past, they are increasingly less useful now, especially in countries such as the UK where large proportions of ethnic minority groups are UK born. Looking forward, there is little alternative to routine recording if we wish to study ethnicity in the long run.

In primary care, we have shown that the recording of valid ethnicity for new patients registering with general practice across the UK has improved dramatically following incentivization under the QOF. The drop-off in ethnicity recording in the final year may be due to the fact that the financial incentivization of ethnicity recording was removed from the QoF scheme for the 2011/12 financial year.

For secondary care, we have shown that the overall completeness of valid ethnicity for HES inpatients has been high for over a decade. Limitations of the remaining HES data sets include poor completeness (for A&E) and poor consistency (for outpatients). The trends shown in the analysis above suggest that completeness of valid coding in these sources is improving. It is possible that prioritizing the recording of valid ethnicity in these settings, perhaps via financial incentivization as in primary care, may facilitate this process. For researchers interested in using routinely recorded ethnicity data, it is important to be aware of the biases that may arise from using incomplete data. The likelihood of having missing ethnicity may not be random, and instead be related to factors such as the circumstances in which patients are admitted, pressures on the available staff and lack of time or opportunity to ask the patient about their ethnicity. As service level factors are not recorded in routine health databases, we cannot estimate the impact these may have on the data.

In order to better understand the patterning of health usage and outcomes for the diverse UK population, we have no choice but to use the ethnicity recorded in routine health databases. Within the CPRD, ethnicity recording among those registered prior to 2006 was low and likely to be highly selective, and thus we recommend not relying on ethnicity from this time period if possible. Since consistency of ethnicity recording is higher in primary care than HES data, when linking the two sources we recommend using primary care ethnicity where available and supplementing with HES ethnicity where required. For patients with multiple ethnicities in any single database, we recommend using the most commonly recorded ethnicity.

### Limitations of this study

This study has not explored ethnicity beyond what has been recorded in routine databases. Though the collection of self-reported ethnicity for all patients is the gold standard across the NHS, it is likely that these data are collected in a range of non-standardized ways across a variety of non-standardized situations. This is a fundamental challenge of using routinely recorded health data for observational studies. Furthermore, due to the nature of this study, we were unable to explore the reasons as to why ethnicity is not recorded, or recorded inconsistently over time and between sources.

## Conclusions

The importance of ethnicity in explaining differences in patterns of disease, health-care usage and outcomes is widely recognized. Previous research has been hampered by deficits in the quality of routine data, and insufficiency of estimation methods. This study has demonstrated that completeness and consistency of routinely recorded ethnicity data in UK-based primary and secondary care have largely improved over time, and with certain caveats, can be usefully incorporated into health research. We have highlighted dramatic improvements in the quality of primary care ethnicity data, particularly since 2006. Completeness of ethnicity information also appears to have been consistently high over the last decade in hospital inpatient settings, but there is still much room for improvement in A&E and outpatient settings. To maximize the value of routinely recorded ethnicity data, both researchers and health-care professionals must work tandem to continuously improve both the quality of these data and their impact via timely research.

## Supplementary data

Supplementary data are available at the *Journal of Public Health* online.

## Funding

This work was supported by a +3 award from the Economic and Social Research Council (ESRC) for Rohini Mathur via the Pathways node of the National Centre for Research Methods. Liam Smeeth is supported by a Wellcome Trust Senior Clinical Fellowship. Krishnan Bhaskaran is supported by a postdoctoral fellowship from the National Institute for Health Research. Emily Grundy is partially supported by the ESRC NCRM Pathways node (grant reference ES/I025561/2). Funding to pay the Open Access publication charges for this article was provided jointly by the Wellcome Trust and the ESRC.

## Supplementary Material

Supplementary Data
