# Supplementary material for: Completeness and usability of ethnicity data in UK-based primary care and hospital databases
Source: J Public Health (Oxf). 2013 Dec 8;36(4):684–92. doi: 10.1093/pubmed/fdt116 (PMC4245896; doi:10.1093/pubmed/fdt116)
Supplement: Supplementary Data [file supp_36_4_684__index.html]

Completeness and usability of ethnicity data in UK-based primary care and hospital databases — Completeness and usability of ethnicity data in UK-based primary care and hospital databases — Supplementary Data 

# Completeness and usability of ethnicity data in UK-based primary care and hospital databases

## Supplementary Data

Supplementary Data

**Files in this Data Supplement:**

- Supplementary Data - Docx file
