# Supplementary material for: Completeness and usability of ethnicity data in UK-based primary care and hospital databases
Source: J Public Health (Oxf). 2013 Dec 8;36(4):684–92. doi: 10.1093/pubmed/fdt116 (PMC4245896; doi:10.1093/pubmed/fdt116)

**Supplementary Materials**

The 16 groups for ethnicity can be further collapsed into five. These are White (British, Irish, other white); south Asian (Bangladeshi, Indian, Pakistani, other Asian); Black (African, Caribbean, black British); Mixed (White and Asian, White and African, White and Caribbean, Other mixed); and other (any other recorded ethnic group).

The 16 groups of the 2001 Census for England and Wales map to the 5 collapsed categories as follows:

| **16 groups** | **5 Categories** |
| --- | --- |
| 1 British | **1.White** |
| 2 Irish |  |
| 3 Any other White background (write in) |  |
| 4 White and Black Caribbean | **2. Mixed** |
| 5 White and Black African |  |
| 6 White and Asian |  |
| 7 Any other mixed background (write in) |  |
| 8 Indian | **3. Asian or Asian British** |
| 9 Pakistani |  |
| 10 Bangladeshi |  |
| 11 Any other Asian background (write in) |  |
| 12 Caribbean | **4. Black or Black British** |
| 13 African |  |
| 14 Any other Black background (write in) |  |
| 15 Chinese | **5. Chinese or Other Group** |
| 16 Any other ethnic group (write in) |  |

**Categorisation of Ethnicity in HES**

| Inpatient 1995-2000 | Inpatient 2001 onwards | Outpatients 2003 onwards and A&E 2008 onwards |
| --- | --- | --- |
| 0 White | A British (White) | An = British (White) |
| 1 Black Caribbean | B Irish (White) | Bn = Irish (White) |
| 2 Black African | C Any other White background | Cn = Any other White background |
| 3 Black Other | D White and Black Caribbean (Mixed) | Dn = White and Black Caribbean (Mixed) |
| 4 Indian | E White and Black African (Mixed) | En = White and Black African (Mixed) |
| 5 Pakistani | F White and Asian (Mixed) | Fn = White and Asian (Mixed) |
| 6 Bangladeshi | G Any other Mixed background | Gn = Any other Mixed background |
| 7 Chinese | H Indian (Asian or Asian British) | Hn = Indian (Asian or Asian British) |
| 8 Any other ethnic group | J Pakistani (Asian or Asian British) | Jn = Pakistani (Asian or Asian British) |
| 9 Not given | K Bangladeshi (Asian or Asian British) | Kn = Bangladeshi (Asian or Asian British) |
| X Not known | L Any other Asian background | Ln = Any other Asian background |
|  | M Caribbean (Black or Black British) | Mn = Caribbean (Black or Black British) |
|  | N African (Black or Black British) | Nn = African (Black or Black British) |
|  | P Any other Black background | Pn = Any other Black background |
|  | R Chinese (other ethnic group) | Rn = Chinese (other ethnic group) |
|  | S Any other ethnic group | Sn = Any other ethnic group |
|  | Z Not stated | Zn = Not stated |
|  | X Not known | X = Not known |

**Table 1a. Proportion of patients registered from April 1^st^ 2006 onwards with matched and mismatched ethnicity in CPRD and HES**

| N=827,753 | Most common code (16 categories) | Most common code (5 categories) |
| --- | --- | --- |
| Matched | 50.6 | 58.6 |
| Mismatched | 49.4 | 41.4 |

**Table 1b. Proportion of patients registered anytime with matched and mismatched ethnicity in CPRD and HES**

| N=3,899,648 | Most common code (16 categories) | Most common code (5 categories) |
| --- | --- | --- |
| Matched | 23.7 | 26.9 |
| Mismatched | 76.3 | 73.1 |

**Table 2. Number of patients in each ethnic category in CPRD and HES**

|  | Most common HES Ethnic Group | | | | | | | |
| --- | --- | --- | --- | --- | --- | --- | --- | --- |
| Most common CPRD Ethnic Group | **White** | **South Asian** | **Black** | **Other** | **Mixed** | **Not Stated** | **Equally common** | **Total** |
| White | 453,244 | 1,294 | 2,082 | 9,549 | 2,095 | 74,714 | 2,271 | 545,249 |
| South Asian | 1,545 | 17,636 | 498 | 3,256 | 11,619 | 6,211 | 1,154 | 41,919 |
| Black | 1,447 | 452 | 1,136 | 1,645 | 21,873 | 4,039 | 822 | 31,414 |
| Other | 2,804 | 4,192 | 271 | 3,392 | 677 | 2,154 | 495 | 13,985 |
| Mixed | 3,487 | 770 | 2,418 | 2,237 | 1,754 | 2,024 | 621 | 13,311 |
| Not Stated | 42,159 | 1,548 | 798 | 1,757 | 2,190 | 7,941 | 373 | 56,766 |
| Equally Common | 1,572 | 667 | 363 | 923 | 1,049 | 819 | 192 | 5,585 |
| Missing | 89,453 | 2,979 | 1,436 | 3,674 | 5,342 | 15,775 | 865 | 119,524 |
| Total | 595,711 | 29,538 | 9,002 | 26,433 | 46,599 | 113,677 | 6,793 | 827,753 |

*Individuals with no ethnicity in either data set are excluded from analysis

| UK Population 5 categories | UK Census 2011 | CPRD census day population crude | CPRD Age standardised | CPRD Cenus day population registered from April 1st 2006 | CPRD Age standardised 2011 population registered from April 1^st^ 2006 |
| --- | --- | --- | --- | --- | --- |
| Population Size | 56,817,259 | 5,219,411 | | 1,446,254 | |
| N with valid ethnicity recorded (%) | | 2,190,797 (42%) | | 1,144,441 (79.1) | |
| White | 87.25% | 86.60% | 86.80% | 82.79% | 84.33% |
| Non White | 12.71% | 13.40% | 13.20% | 17.21% | 15.67% |
| Mixed | 1.97% | 1.29% | 1.27% | 1.87% | 1.58% |
| South Asian | 6.18% | 5.95% | 5.80% | 7.60% | 6.78% |
| Black | 2.98% | 3.73% | 3.74% | 4.46% | 4.27% |
| Other | 1.57% | 2.43% | 2.39% | 3.27% | 3.04% |
| *CPRD Excludes those who have no ethnicity or whose last recorded ethnicity is not stated | | | | | |

| UK Population 16 categories (condensed) | UK Census 2011 | CPRD census day population crude | CPRD Age standardised | CPRD Cenus day population registered from April 1st 2006 | CPRD Age standardised 2011 population registered from April 1^st^ 2006 |
| --- | --- | --- | --- | --- | --- |
| *Population Size* | 56,817,259 | 5,219,411 | | 1,446,254 | |
| N with valid ethnicity recorded (%) | | 2,190,797 (42%) | | 1,144,441 (79.1) | |
| White British/Scottish/Welsh/Irish | 83.17% | 70.59% | 71.01% |  | **-** |
| Other White | 4.07% | 16.01% | 15.79% |  | - |
| *White (All)* | ***87.25%*** | ***86.60%*** | ***86.80%*** | ***65.32%*** | ***67.73%*** |
| Chinese | 0.66% | 0.56% | 0.55% | 17.24% | 16.60% |
| Indian | 2.3% | 2.27% | 2.19% | 82.56% | 84.33% |
| Pakistani | 1.8% | 1.34% | 1.32% | 0.80% | 0.77% |
| Bangladeshi | 0.7% | 0.48% | 0.47% | 2.77% | 2.41% |
| Other Asian | 1.3% | 1.86% | 1.82% | 1.68% | 1.45% |
| Black Caribbean | 0.9% | 0.77% | 0.79% | 0.62% | 0.53% |
| Black African | 1.6% | 1.88% | 1.86% | 2.58% | 2.39% |
| Black Other | 0.4% | 1.08% | 1.09% | 0.79% | 0.79% |
| Mixed | 2.0% | 1.29% | 1.27% | 2.56% | 2.26% |
| Other | 0.9% | 2.43% | 2.39% | 1.36% | 1.22% |
| *CPRD Excludes those who have no ethnicity or whose last recorded ethnicity is not stated | | | | | |

**Figure 1a. Proportion of patients in CPRD with any ethnicity code recorded in the same financial year as first registration with the general practitioner by gender.**

**Figure 1b. Proportion of patients in CPRD with any ethnicity code recorded in the same year as first registration with the general practitioner by age at registration.**

**Figure 2. Regional breakdown of patients contributing to CPRD and the 2011 UK Census on March 20^th^ 2011**

**Figure 3a. Comparison of age structure in the 2011 UK Census Population and 2011 CPRD Population**

**Figure 3b. Age structure in the 2011 UK Census Population and 2011 CPRD Population by gender on March 27th, 2011**

**Figure 3c. Age Structure of Census day CPRD population by ethnic group on March 27th, 2011**

Figure 4a. Ethnicity recording for all patients in the July 2012 Build of CPRD


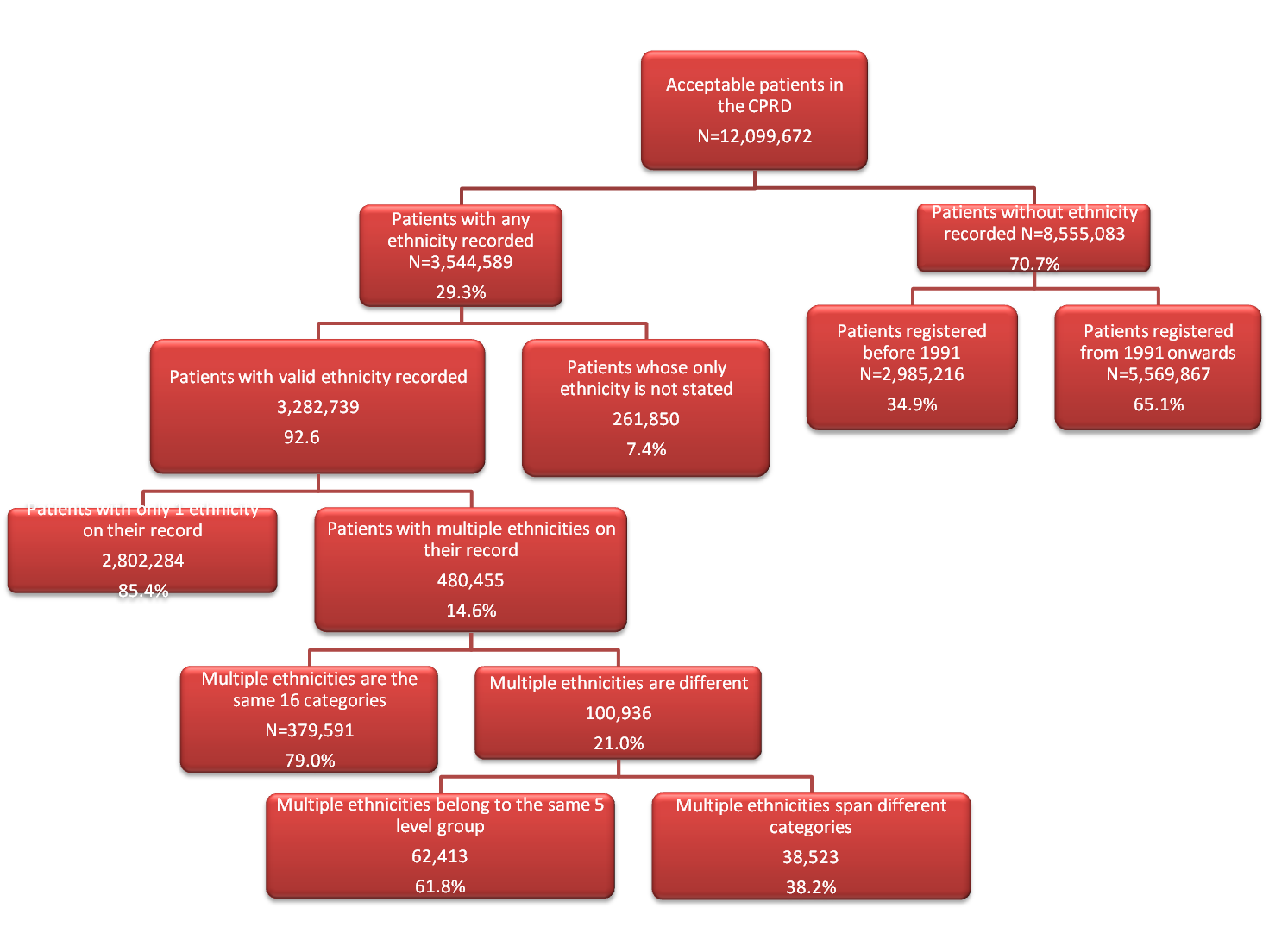


Figure 4b. Ethnicity recording for all currently registered patients in the July 2012 Build of CPRD


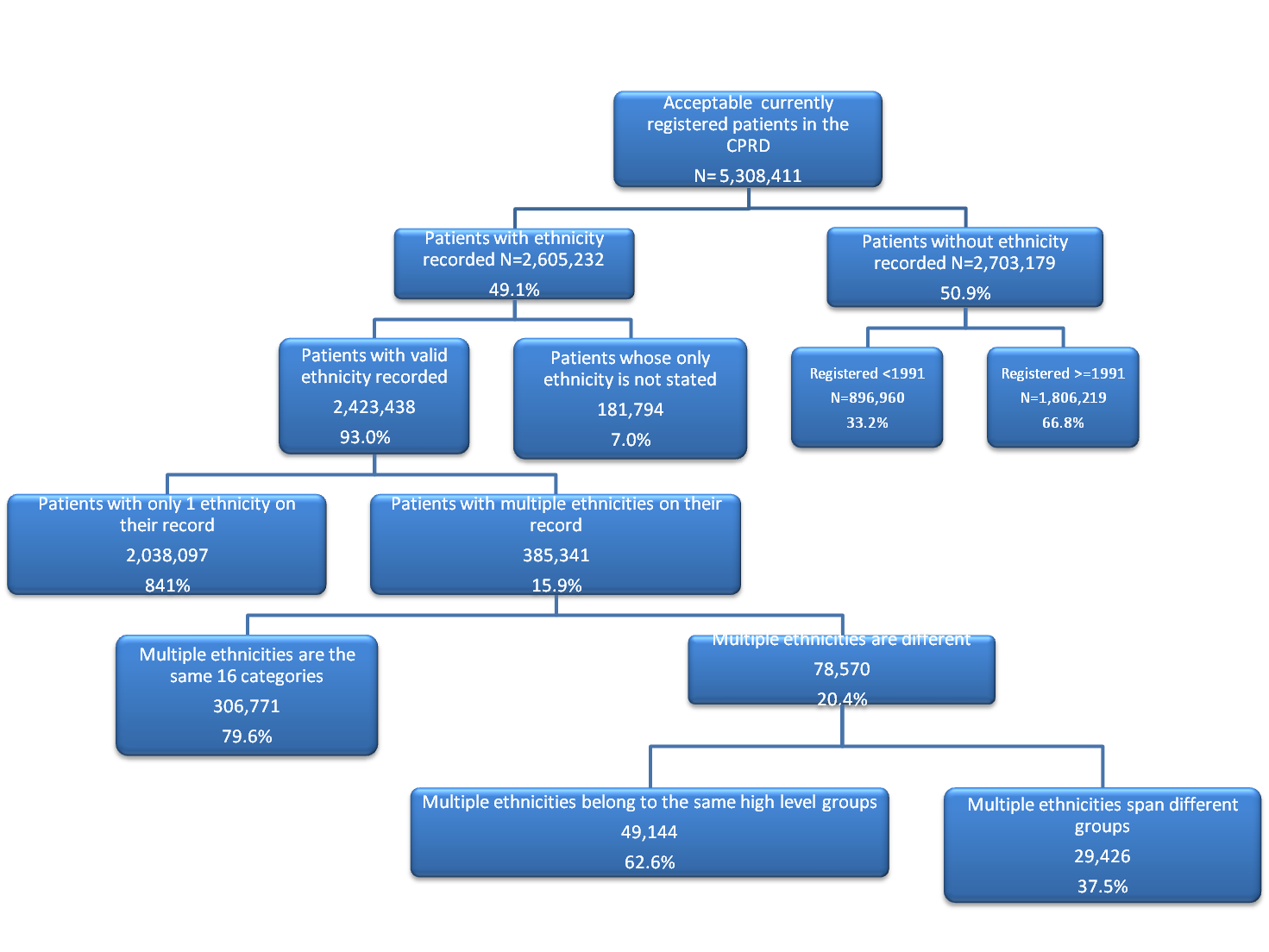


Figure 4c. Ethnicity recording for all patients registered from April 1^st^ 2006 in the July 2012 Build of CPRD


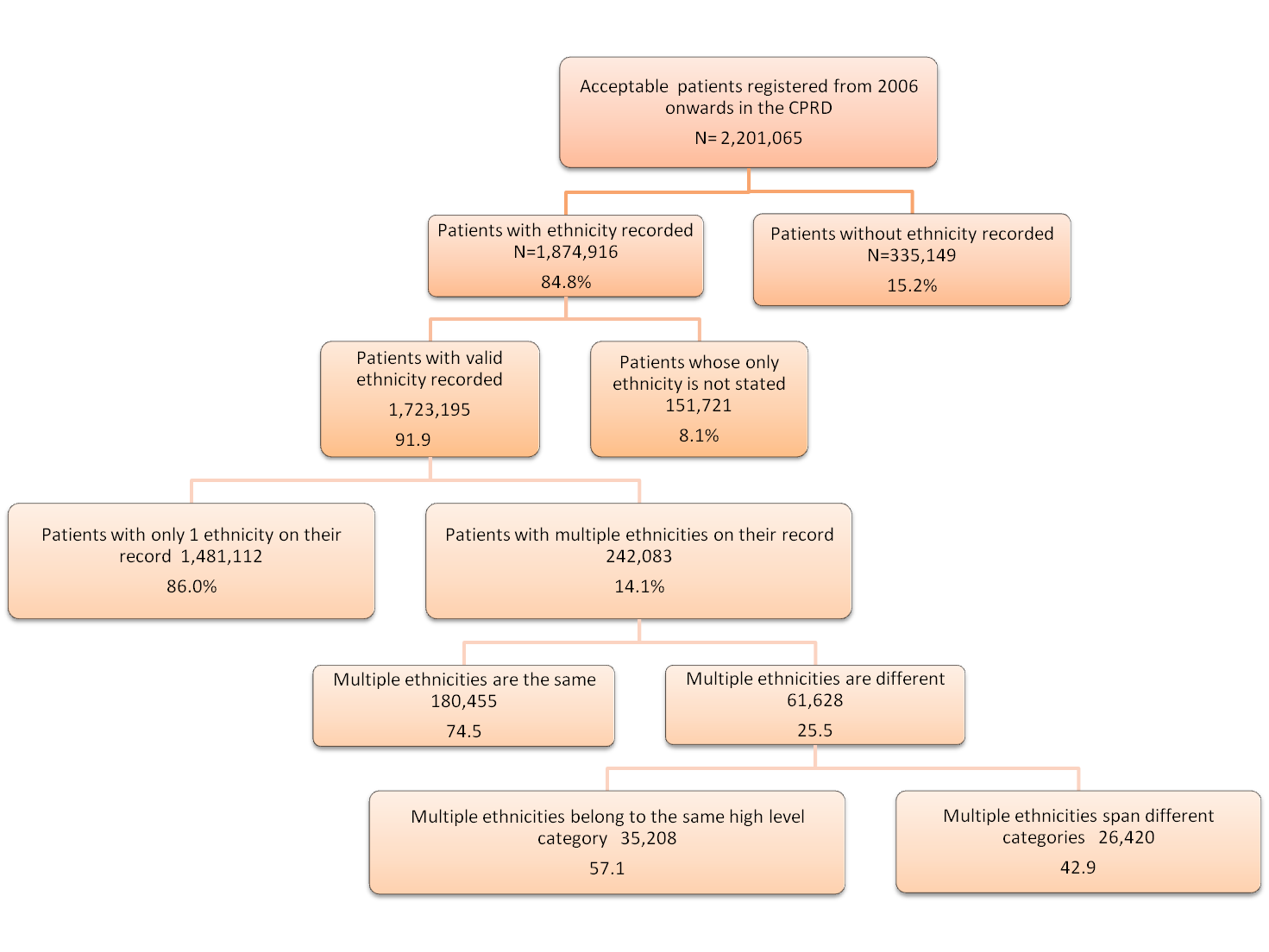


Figure 5a. Ethnicity recording for all HES inpatients as of April 2012


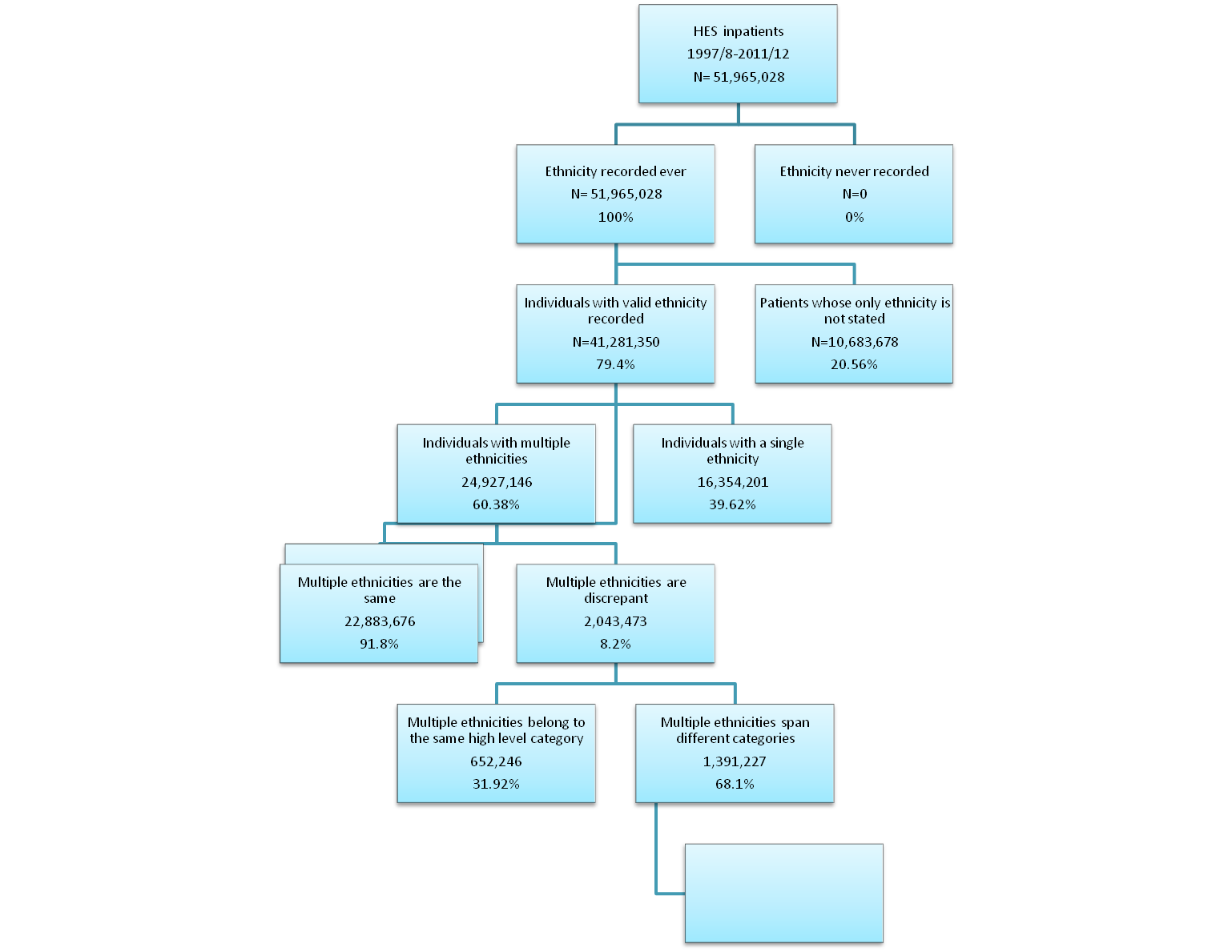


Figure 5b. Ethnicity recording for all HES Outpatients as of April 2012


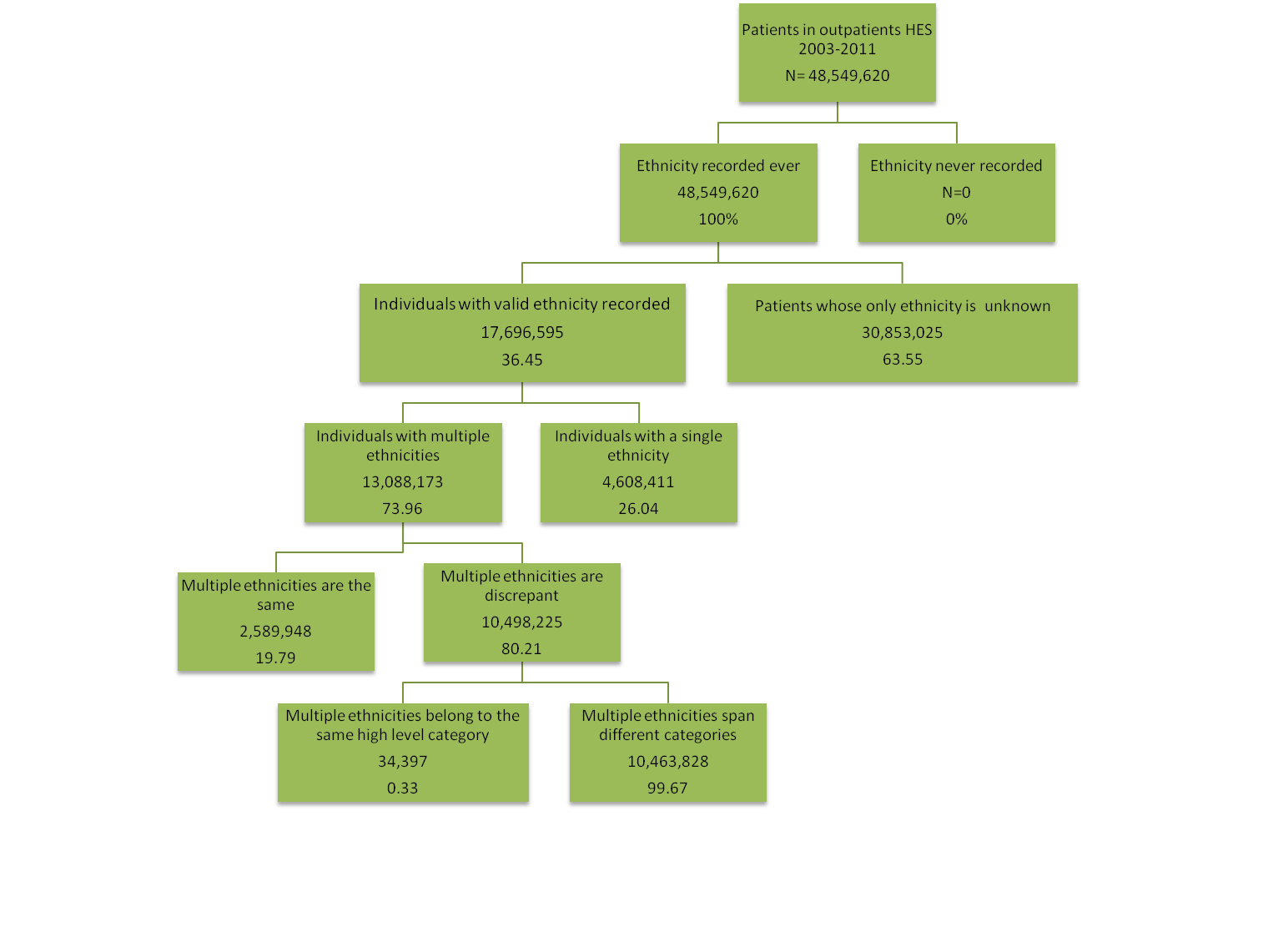


Figure 5c. Ethnicity recording for all HES A&E patients as of April 2012


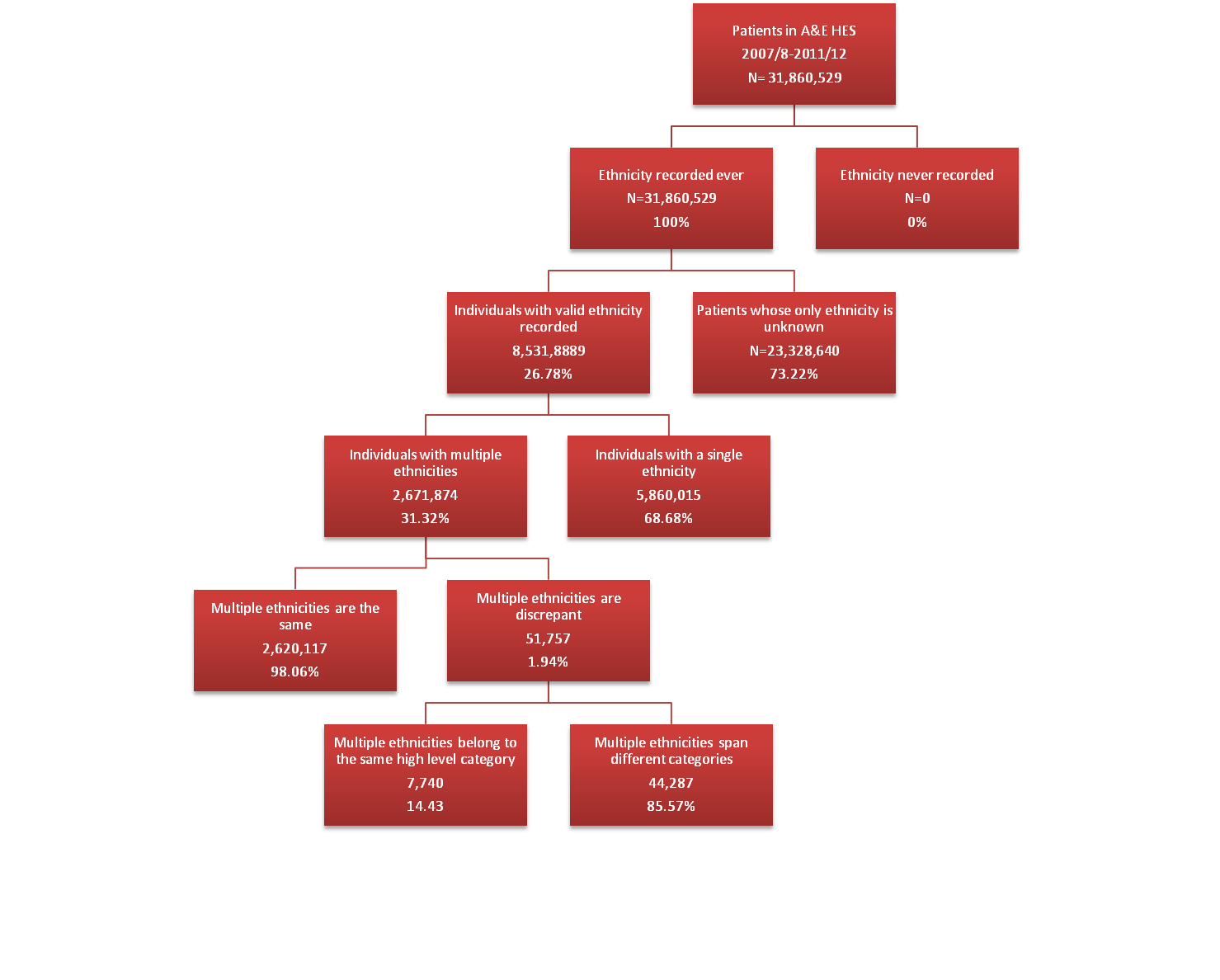

Supplement: Supplementary Data [file supp_fdt116_fdt116supp.docx]
